# Characterizing Heparin Tetrasaccharides Binding to Amyloid-Beta Peptide

**DOI:** 10.3389/fmolb.2022.824146

**Published:** 2022-02-23

**Authors:** Xiang Zhou, Yuanyuan Wang, Wei Zheng, Guangxiu Deng, Fuyi Wang, Lan Jin

**Affiliations:** ^1^ National Glycoengineering Research Center, Shandong Key Laboratory of Carbohydrate Chemistry and Glycobiology, Shandong University, Qingdao, China; ^2^ Beijing National Laboratory for Molecular Sciences, CAS Key Laboratory of Analytical Chemistry for Living Biosystems Institute of Chemistry, Chinese Academy of Sciences, University of Chinese Academy of Sciences, Beijing, China; ^3^ College of Traditional Chinese Medicine, Shandong University of Traditional Chinese Medicine, Jinan, China

**Keywords:** heparin, β-amyloid peptide, interaction, NMR, hydrogen/deuterium exchange mass spectrometry

## Abstract

The aggregation of β-amyloid peptide (Aβ) is one potential cause for Alzheimer’s disease (AD). Heparin can either promote or inhibit Aβ aggregation. The sulfation pattern and chain size determine its binding affinity and its role. Using 2D-NMR analysis and molecular modelling, the binding motif of heparin oligoaccharides to Aβ was determined to be HexA-GlcNS-IdoA2S-GlcNS6S. Iduronic acid epimerization and 6-O-sulfation are key factors for the binding affinity, while 3-O-sulfation of Arixtra (heparin pentasaccharide) is not involved in the binding to Aβ. Hydrogen-deuterium exchange mass spectrometry (HDX-MS) was used to study the glycosaminoglycan (GAG)-peptide complex and identified V12HHQKL17 as the binding site of GAG at Aβ. Furthermore, an MTT assay was applied to evaluate the anti-Aβ fibril formation function of heparin tetrasaccharide, and indicated that the heparin tetrasaccharide with the defined sequence represents a promising inhibitor of Aβ aggregation.

## 1 Introduction

Alzheimer’s disease (AD) is a neurodegenerative disease that mainly occurs in people over 65 years of age. The number of patients suffering from AD around the world was 46.8 million in 2015, a number expected to increase rapidly to approximately 131.5 million in 2050. The currently available treatments for AD are post-symptomatic and provide limited relief. In recent years, amyloid plaques and Tau pathology have been widely accepted as the two hallmarks of AD, and their explicit mechanisms lead to the development of therapies that target the course of the disease. The amyloid plaques are mainly composed of β-amyloid peptide (Aβ), which is derived from the amyloid precursor protein and consists of 39–42 amino acid residues (see Supplementary Figure S4 in the Supplementary Material). Under physiological conditions, different forms of Aβ, including the nontoxic monomer, toxic and soluble oligomers, and aggregated amyloid fibrils, co-exist in a dynamic equilibrium (Benilova et al., 2012). An excess of Aβ can push the equilibrium to amyloid fibrils, and the fibrils then infringe the nerve cells and potentially increase the risk of AD. Several immunotherapeutic antibodies targeting Aβ have reached phase II or III clinical trials (Ariga et al., 2010). Several AD related drugs have also been submitted to the Center for Drug Evaluation worldwide. One of them is Aducanumab (Biogen), which has been approved controversially by FDA but rejected by EMA this year, due to its uncertainty of the efficacy. In addition to these protein-based drugs, sulfated oligosaccharides are promising agents to neutralize excess Aβ because they are less costly and capable of passing the blood-brain barrier. However, carbohydrates are usually heterogeneous and can bind to many proteins. Their undefined structures and unspecific mechanisms are major challenges for developing carbohydrate-based drugs. Heparin related oligosaccharides are being actively investigated as potential drugs for the treatment of AD, which is possibly the result of the binding between the sulfated oligosaccharides and Aβ. However, the detailed binding mechanism between the sulfated oligosaccharides and Aβ has not been elucidated.

Heparin belongs to the glycosaminoglycan (GAG) family, which includes heparan sulfate (HS), chondroitin sulfate (CS), dermatan sulfate, hyaluronic acid and other subfamilies based on their different repeating disaccharide units and sulfation patterns. GAGs can either promote or inhibit the aggregation of Aβ, with the size of the GAG molecules being the key factor. For example, HS polysaccharide accelerates Aβ aggregation, while small heparin oligosaccharides can inhibit and reverse the process of aggregation. (Zhu et al., 2001; Walzer et al., 2002). The reason is that the large GAG can bind to multiple Aβ monomers and act as a core for the aggregation, while the small oligosaccharides can only bind to single Aβ monomers and hinder the formation of Aβ β-sheet or peeling Aβ from the fibrils. The negatively charged groups on GAGs are important because of their binding affinity to Aβ. For example, when the sulfo groups were removed from HS, it lost its ability to promote Aβ fibril formation (Castillo et al., 1999). Binding of Aβ to heparin/HS oligosaccharides has been studied using solid-state NMR. There were no significant differences of the K_d_ values (∼40 μM) from hexamer to octadecamer derived from heparin degradation (Stewart et al., 2016). N-sulfation or 6-O-sulfation in chemically synthesized HS polysaccharides was essential in binding with Aβ beyond electrostatic attraction (Madine et al., 2012). Study of HS hexasaccharide and decasaccharide obtained by well-defined synthesis in binding with isotopically labeled Aβ indicated the importance of specific amino acids at the C-terminus residues in the Aβ fibrils (Wang et al., 2021). CS disaccharides with different sulfation positions have been shown different binding forces to Aβ, with an ordering from strong to weak of 6-O-sulfation, 4-O-sulfation, and 2-O-sulfation (Fraser et al., 2001). Conformation of proteins and their interaction with carbohydrates were studied more and more using HDX-MS (Chalmers et al., 2011; Engen, 2009). In this study, we elaborate the roles of each sulfo and carboxyl group of heparin tetrasaccharides and delineate the sequence required for binding to Aβ.

## 2 Materials and Methods

### 2.1 Materials

Heparin was purchased from Zaozhuang Sinock Biochemical Pharmaceutical Co., Ltd. Heparinase I was purchased from Beijing Adhoc International Technologies Co., Ltd. Fondaparinux injection was obtained from the hospital. Aβ was solid synthesized by ChinaPeptides Co., Ltd. Deuterium oxide (99.9% D_2_O, CIL) was purchased from Qingdao Tenglong Weibo Technology Co., Ltd. Norell S-5-500-HW-7 NMR tubes were used for the complex samples of heparin oligosaccharides with Aβ. Normal 5 mm NMR tubes were used for the oligosaccharide structure characterization.

### 2.2 Methods

#### 2.2.1 Preparation of Heparin Tetrasaccharides

Heparin was partially digested with heparinase I. The oligosaccharides were fractionated using a GE AKTA purifier 100 system with a Superdex TM Peptide 10/300 GL column. The fractions were eluted with 0.2 M NH_4_HCO_3_ at a flow rate of 0.4 ml/min. The tetrasaccharide fraction was then separated using a Spherisorb S5 SAX 5 μm column. A linear gradient of 0–2.0 M NaCl (pH 3.5) over 2 h was used. The flow rate was 4 ml/min. UV absorption was monitored at 232 nm. ^1^H spectra were used to characterize the purity based on the signal integration.

#### 2.2.2 Structure Characterization of Oligosaccharides by MS/MS

MS/MS studies were performed on an ion-trap-time-of-flight (IT-TOF) hybrid mass spectrometer (Shimadzu) in the negative mode. The samples were injected using a syringe in 50:50 (v:v) methanol/water containing 1 mM NaOH. The instrument parameters were set as follows: interface voltage, −3.5 kV; nebulizing gas flow rate, 1.5 L/min and collision-induced dissociation energy: 50%. The sequence was elucidated by glycosidic fragmentations and cross-ring fragmentations.

#### 2.2.3 Structure Characterization of Oligosaccharides by 2D-NMR

3 mg of each oligosaccharide was dissolved in 500 μL of deuterium oxide (D_2_O) and lyophilized to remove exchangeable protons. The powder was then re-dissolved in 550 μL of D_2_O and transferred into 5 mm NMR tubes. NMR spectra were acquired at 600 MHz (^1^H) or 150 MHz (^13^C) with a Bruker AVANCE III 600 MHz spectrometer equipped with a 5 mm cryoprobe. Chemical shifts were recorded with reference to the HDO solvent signal at 298 K. 2D ^1^H-^1^H COSY, ^1^H-^13^C HSQC, and ^1^H-^13^C HMBC were acquired with standard pulse sequences. Cross peaks in the HSQC spectra of each oligosaccharide were assigned and labeled.

#### 2.2.4 Molecular Docking and Modeling Simulations

Molecular docking and modeling were performed using AutoDock 4.2.6, a fully automatic docking program available open source. The solid-state NMR structure of Aβ was retrieved from the Protein Data Bank (PDB) under code 6TI5 (Cerofolini et al., 2020), which is a fibril of hexadecylmer, while an NMR-optimized structure was used for tetrasaccharide 1 (Jin et al., 2009). All of the hydrogen atoms were added to Aβ fibril and Gasteiger charge was used. A volume of (120, 120, 120) grid points with 0.514 Å spacing was used. Aβ was put in the center of the cube larger enough to cover the whole surface of Aβ. The tetrasaccharide **1** was placed randomly into the box. During the whole docking process, all monosaccharide rings were fixed at their starting conformations, while the ring substituents were defined as flexible and could be rotated freely. Genetic Algorithm was used for docking.

#### 2.2.5 GMSA Assay

The GMSA assay was performed according to procedures previously described. Briefly, 1 mg Aβ was dissolved in 500 μL NaAc (20 mM, pH 3.5, precooled in ice) without any disaggregation process and the final concentration of 2 μg/μL Aβ was used thereafter. 2-aminoacridone-labeled tetrasaccharides were combined with Aβ in a certain molar ratio for 15 min at room temperature in a total volume of 20 μL. They were then loaded in wells of 1% agarose gel in 10 mM Tris-HCl (pH 7.4) and 1 mM EDTA. Electrophoresis was performed at 100 V for 20 min in a horizontal agarose electrophoresis system using an electrophoresis buffer comprising 40 mM Tris/acetic acid (pH 8.0) and 1 mM EDTA. The fluorescent oligosaccharides were visualized on a FluorChem Q gel analysis system.

#### 2.2.6 HDX-MS Analysis

An in-house system was set up using a Waters Acquity-H-CLASS UPLC and Xevo G2 Q-TOF mass spectrometer. A refrigerator was used to cool the injector, loop, columns, 6-way valve and switch valve. Aβ was treated in TFA and HFIP to break up any possible preexisting aggregates. The complex of Aβ and tetrasaccharide 1 was incubated in H_2_O (pH 3.5) at 4°C for 120 min. Nine volumes of D_2_O (pH 3.5) were added to initiate the HDX. After 10 min, 1 μL of 10% ice-cold formic acid (FA) solution (pH 2.5) was added to quench the HDX. The deuterated Aβ was loaded onto an on-line pepsin proteolytic column. After digestion, the valve was switched, and the peptides flowed into a C18 column (5 μm, 2.1 × 50 mm). The mobile phase A was 0.1% FA, and mobile B was 0.1% FA in acetonitrile. The elution gradient was 20–60% mobile phase B in 6 min. The flow rate was 200 μL/min. The mass spectrometer parameters were set as follows: capillary voltage, 3 kV; source temperature, 100°C; desolvation temperature, 350°C. The spectra were acquired in MSe mode with low collision energy of 6 V and high collision energy of 25–50 V.

#### 2.2.7 MTT Assay

The SH-SY5Y and PC 12 cell lines were cultured separately in Dulbecco’s modified Eagle’s medium supplemented with 10% fetal bovine serum at 37 °C, 5% CO_2_ in an incubator. The cells (4,000 or 6,000 cells/well) were seeded in 96-well plates and cultured in DMEM with 10% FBS. The culture medium was changed to fresh medium supplemented with 2 μM Aβ peptide alone or with tetrasaccharide 1 at concentrations of 10, 50 or 100 μg/ml. After 24 or 36 h incubation, 20 μl of 5 mg/ml MTT was added. After 4 h incubation at 37°C, the supernatant was discarded, and 150 μL of DMSO was added. Enzyme-linked immunoassay was used to measure the absorbance value at 570 nm for the determination wavelength and 630 nm for the reference wavelength. Statistical significance was established by SPSS 17.0 statistical software, and single factor analysis of variance and *p*-values were represented as follows: *p < 0.05 and **p < 0.01. The experimental results were in the form of the mean and standard deviation.

#### 2.2.8 Interaction of Aβ With Heparin Oligosaccharides

Heparin oligosaccharide was dissolved in D_2_O, into which Aβ was added, and the supernatant was transferred into the NMR tube. Complexes of heparin oligosaccharides and Aβ were prepared with a molar ratio of 1:1 in 120 μL of D_2_O. ^1^H-^13^C HSQC spectra were acquired.

## 3 Results

### 3.1 Preparation of Heparin Tetrasaccharides

Three heparin tetrasaccharides **1-3** with different sulfation patterns were prepared by partial enzymatic digestion of heparin, gel permeation chromatography fractionation, and strong anion-exchange liquid chromatography separation (Supplementary Figure S1). ^1^H spectra were used to characterize the purity based on the signal integration. (Supplementary Figure S3). The impurities were labelled as asterisks and the amount of which were up to 12.2%.

### 3.2 Structure Characterization of Oligosaccharides by MS/MS and 2D-NMR

**FIGURE 1:**
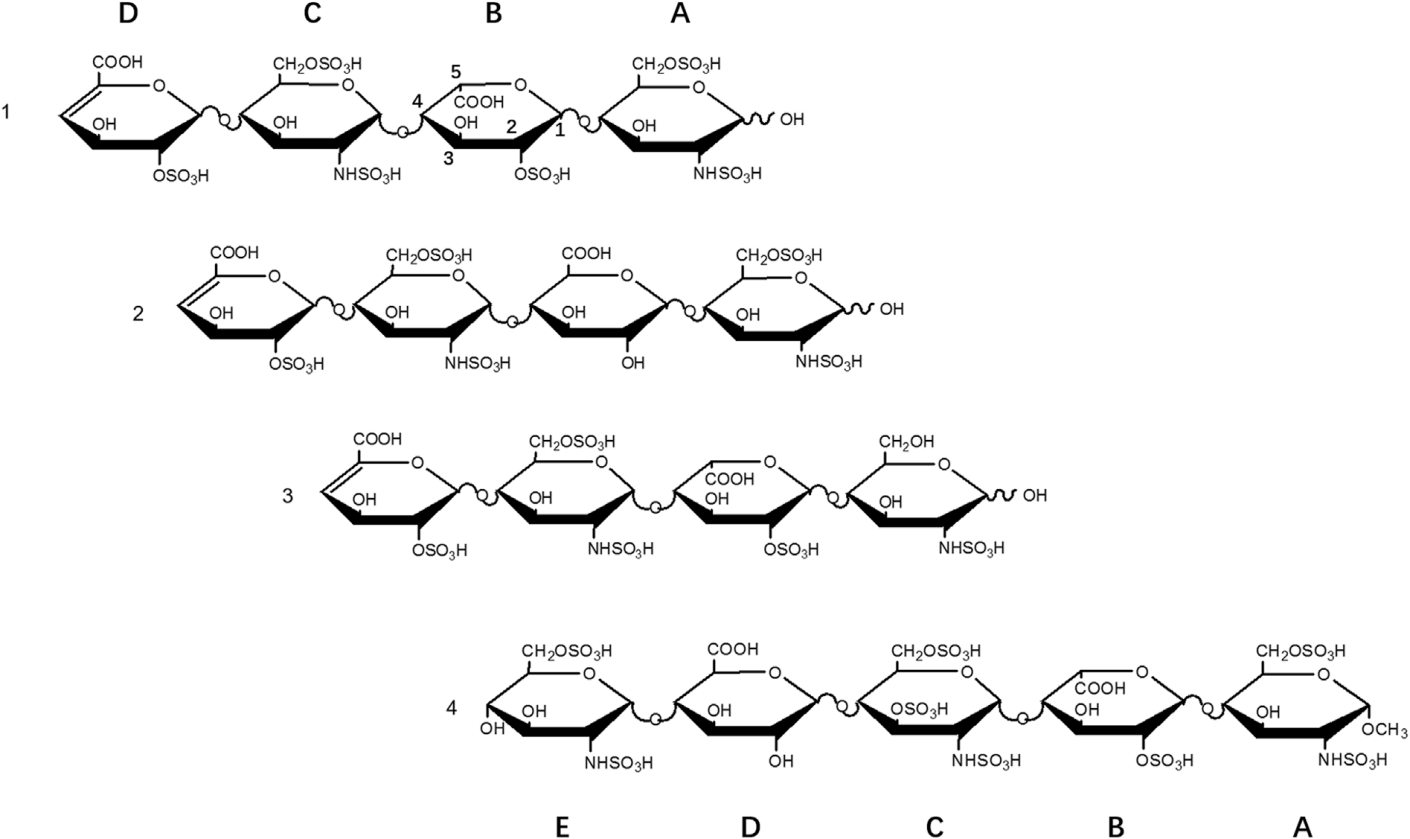
Structures of heparin oligosaccharides.

The structures were determined by tandem MS and 2D-NMR (Supplementary Figures S2, S4). Tetrasaccharide 1, ΔUA2S-GlcNS6S-IdoA2S-GlcNS6S, is the fully sulfated form, i.e., substituted by three sulfo groups per disaccharide. Tetrasaccharide 2, ΔUA2S-GlcNS6S-GlcA-GlcNS6S, is the 2-O-desulfated and epimerized form of 1, and tetrasaccharide 3, ΔUA2S-GlcNS6S-IdoA2S-GlcNS, is the 6-O-desulfated form of 1 (Figure 1).

### 3.3 Molecular Docking and Modeling Simulations

**FIGURE 2:**
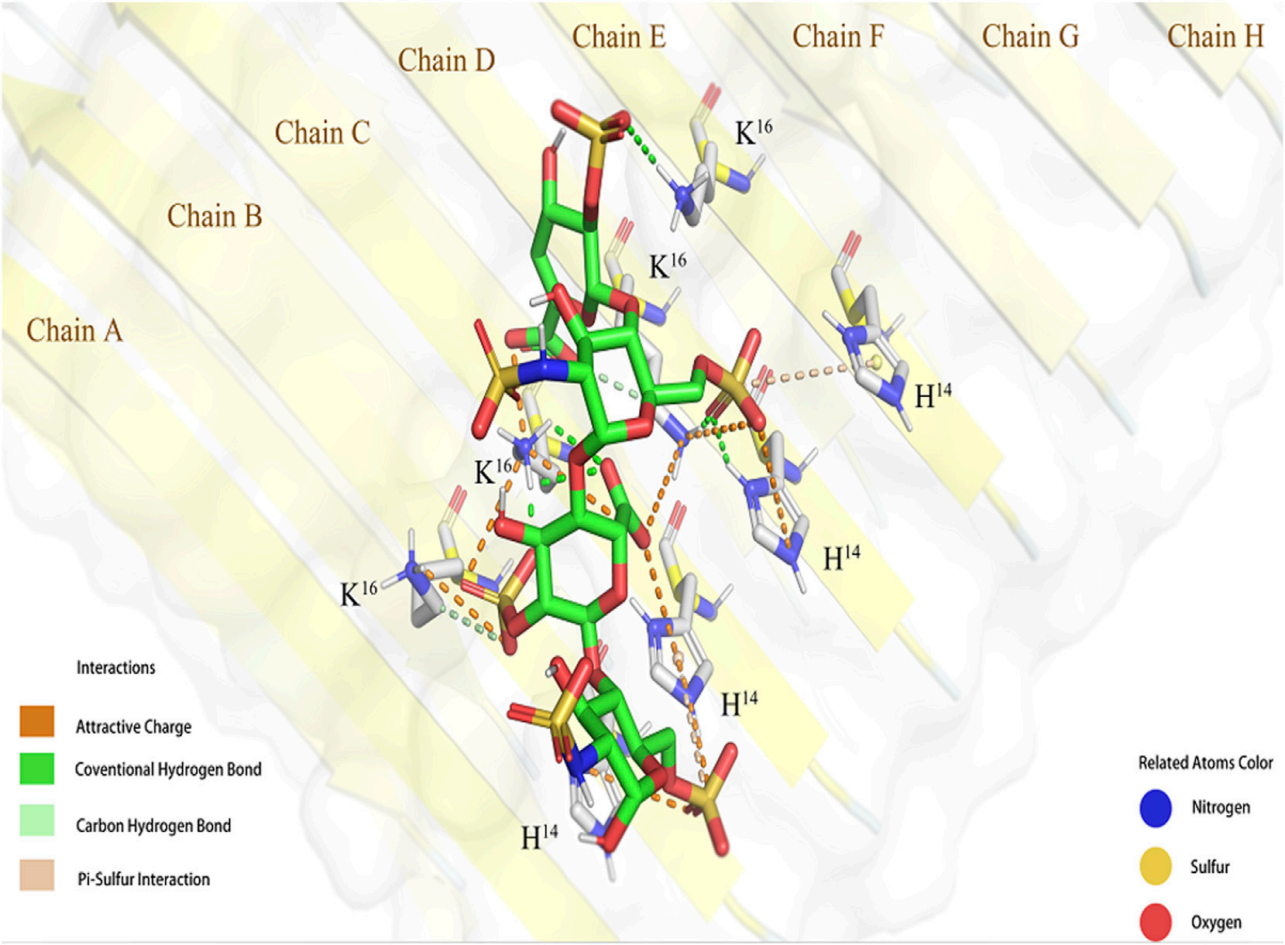
Simulated interaction between **1** and Aβ fibril (6TI5). The conformation of **1** and Aβ binding complex generated by molecular docking shown in Pymol.

The conformation with the lowest binding energy (Figure 2) was examined using the Pymol software to evaluate the degree of ionic interaction between specific sulfo and carboxyl groups of tetrasaccharide 1 and the basic amino acid residues of Aβ. The Aβ fibril was a hexadecylmer in an anti-parallel construction, composed by eight chains (A-H chains) on each side. And four chains (B-E) interacted with one tetrasacchride 1 mainly through ionic attraction and hydrogen bonds. These interactions involved all His-14 and Lys-16 in B-E chains.

### 3.4 GMSA Assay

**FIGURE 3:**
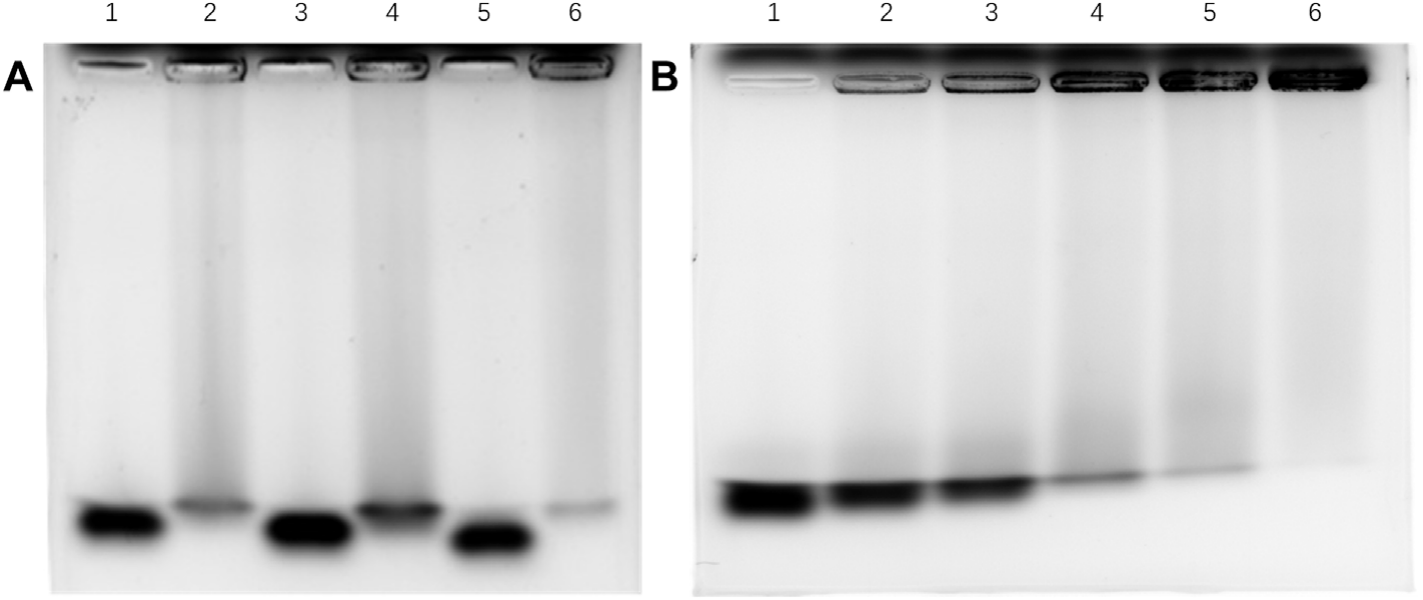
Binding affinity between Aβ and **1**-**3** by GMSA assay **(A)** Lane 1: **3** alone; lane 2: **3** and Aβ in the ratio of 1:2; lane 3: **2** alone; lane 4: **2** and Aβ in the ratio of 1:2; lane 5: **1** alone; lane 6: **1** and Aβ in the ratio of 1:2; **(B)** Lane 1: tetrasaccharide mixture alone; lanes 2–6: tetrasaccharide mixture and Aβ in the ratios of 1:1, 1:2, 1:3, 1:4 and 1:6, respectively.

A gel mobility shift assay (GMSA) was used to evaluate the binding affinities of tetrasacchrides 1-3 to Aβ containing 40 amino acid residues. The tetrasaccharides were derived by 2-aminoacridone, incubated with Aβ, co-electrophoresed, and detected by fluorescence on a FluorChem Q gel analysis system (Figure 3). The tetrasaccharide-Aβ complexes showed slower motilities than their corresponding controls of tetrasaccharide alone. Tetrasaccharide 1 showed the strongest binding affinity, as most of it was withheld by Aβ at the sample loading position, and only a faint band corresponding to the unbound format was observed. Tetrasaccharide 3 exhibited much weaker binding affinity than 1, while tetrasaccharide 2 bonded most weakly to Aβ.

### 3.5 Interaction of Aβ With Heparin Oligosaccharides

**FIGURE 4:**
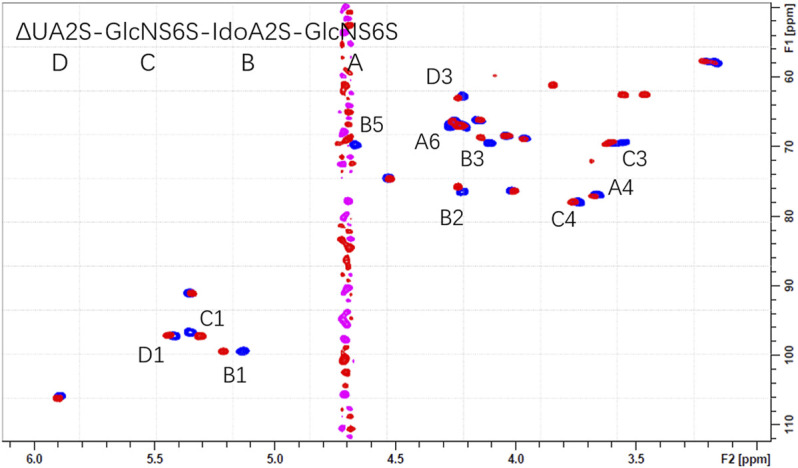
The overlaid ^1^H-^13^C HSQC spectra of **1** only (blue) and **1** with Aβ (pink).

The overlaid HSQC spectra of all oligosaccharides 1-4 only and with Aβ were shown in Supplementary Figure S6. Obvious chemical shift perturbations of certain cross peaks upon the binding were observed after the addition of Aβ. The results indicated that the 3-O-sulfo group did not contribute to the binding but did decrease the affinity, which could be due to the stereo-hindrance effect. (Figure 4 and Supplementary Figure S6).

### 3.6 HDX-MS Analysis

**FIGURE 5:**
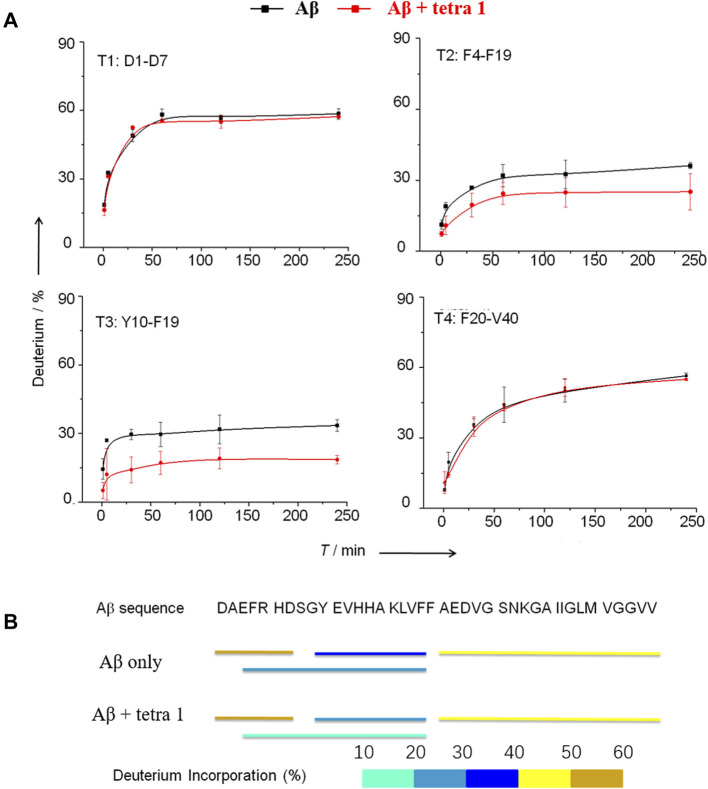
HDX-MS analysis of Aβ showing significant decrease in the HDX rate of amino acids residues Tyr10—Phe19 in A upon binding to **1**. **(A)** The deuterium exchange rate (%) of four peptic peptides deriving from Aβ incubated in D_2_O for various time in the absence and the presence of 1. T1 = D1AEFRHD7; T2 = F4ARHDSGYEVHHQKLVEF10; T3 = Y10EVHHQKLVF19; T4 = F20AEDVGSNKGAIIGLMVGGVV40. **(B)** Deuterium in-corporation (%) of peptic peptides arising from Aβ without and with binding to **1** after incubated in D_2_O buffer for 4 h.

The deuterated Aβ was loaded onto an on-line pepsin proteolytic column for digestion. Four proteolytic peptides, designated T1, T2, T3, and T4, derived from peptic digestion of Aβ were detected to cover the full sequence of Aβ (Figure 5). The changes of deuterium incorporation for each peptic peptide subject to incubation of Aβ in D2O for different times were monitored by MS. The data were analyzed by HX-Express software based on the centroid of the molecular ion isotope peaks.

### 3.7 MTT Assay

**FIGURE 6:**
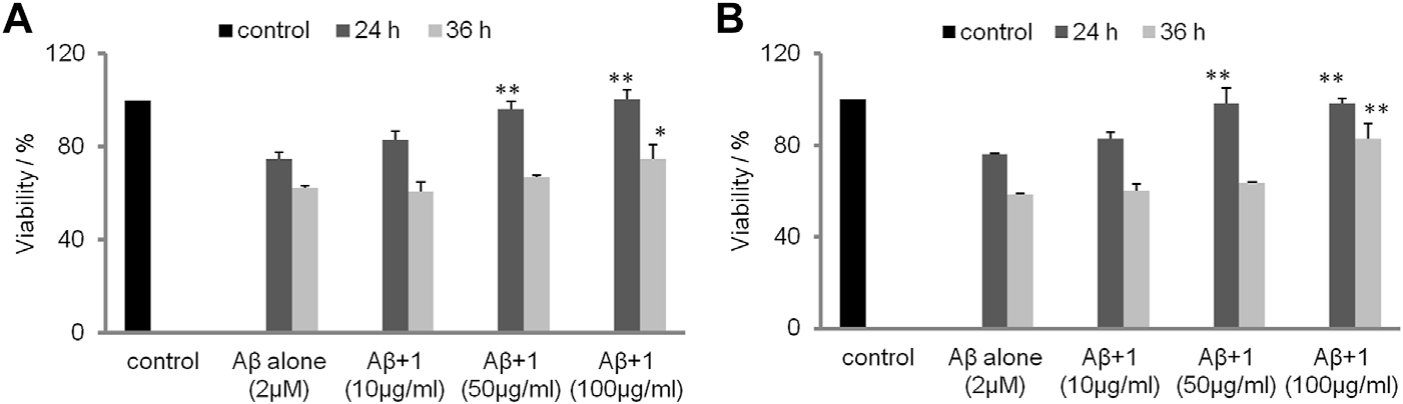
The inhibition effect of **1** against the cytotoxicity caused by Aβ. **(A)** SH-SY5Y cells and **(B)** PC12 cells after 24 and 36 h (*p < 0.05, **p < 0.01).

The inhibitory activities of tetrasaccharide 1 against Aβ fibril formation were assessed by an MTT assay using neurocyte cell lines SH-SY5Y and PC12, respectively (Figure 6). The viability of neurocytes decreased significantly after exposure to 2 µM aged Aβ for 24 and 36 h, respectively. The toxicity of Aβ was attenuated when tetrasaccharide 1 was added to the cells. Addition of tetrasaccharide 1 at 100 μg/ml could significantly reduce the cell injuries caused by Aβ. This effect indicated that the interaction between tetrasaccharide 1 and Aβ can inhibit the formation of the toxic oligomers or aggregates.

## 4 Discussion

The negatively charged groups on the GAG oligosaccharides are responsible for its affinity to Aβ. The sulfo groups of heparin and HS can be sorted to 2-O-sulfo of the uronic acid residue, 3- and 6-O-sulfo at the GlcN residue, and N-sulfo of the GlcN residue. GMSA results suggest that the 2-O-sulfo group at the uronic acid residue and 6-O-sulfo group at the terminal residue are involved in the interaction with Aβ. The docking simulation suggested that the His-14 to Lys-16 regions of Aβ fibril (B-E chains) were involved in binding with tetrasaccharide 1. The IdoA2S residue was in the ^1^C_4_ conformation, and both carboxyl group and 2-O-sulfo group were in closer contact with Aβ. IdoA2S was electrostatically attracted by His-14 (C chain) and Lys-16 (B-D chains) of Aβ, and was the essential motif in binding. It is worth mentioning that the 6-O-sulfo group at the reducing end is attracted by His-14 in the B and C chains of Aβ, which explains why tetrasaccharide 2 also bonded to Aβ to some degree in the GMSA experiment.

In NMR analysis, the synthetic Aβ served as a probe to elucidate the essential binding regions of 1. As shown in Figure 4, GlcNS (A2, C1, C2, C3), 6S at the reducing end (A6S), IdoA2S (B1, B2, B3, B5) and the carboxyl group of the non-reducing end (D4) were crucial to the binding. Obvious chemical shift perturbations of certain cross peaks upon the binding were observed after the addition of Aβ. The internal monosaccharide rings -GlcNS6S-IdoA2S- (C-B) were involved in the binding interaction, with the IdoA2S showing the largest chemical shift perturbations, suggesting that it plays an essential role in binding with Aβ. By combining the molecular docking and HSQC-NMR results, the binding sequence for heparin tetrasaccharides to bind to Aβ could be deduced to be HexA-GlcNS-IdoA2S-GlcNS6S. The conformation of the internal hexauronic acid residue and the proper orientation of the substituents were the determinants. In the linear structure of heparin, in addition to the 2-O-sulfation at the uronic acid residue and 6-O-sulfation and N-sulfation at the GlcN residue included in 1, the 3-O-sulfation at the GlcN residue occasionally occurs and is responsible for the anticoagulant activity of heparin. The 3-O-sulfation in Arixtra (4 in Figure 1) did not contribute to the binding with Aβ based on the HSQC spectrum, but IdoA2S was still involved in binding. And this could be due to the stereo hindrance effect.

Based on the HDX-MS results, when 1 was present, the peptides T2 and T3 showed a significant decrease of deuterium update rate, while the rates of peptides T1 and T4 remained almost unchanged (Figure 5). These results provide evidence that the Tyr10-Phe19 region participates in the interaction between Aβ and 1, in consistent with prediction by docking simulation. Cardin and Weintraub (Cardin and Weintraub, 1989) proposed a consensus sequence XBBXBX (where B is a basic amino acid residue and X is any amino acid residue) in proteins to be the binding site to GAGs. Nguyen (Nguyen and Rabenstein, 2016) confirmed the binding affinity of Aβ12-18 fragment to heparin decreased dramatically if the amino acid residue His-13 or His-14 was replaced by alanine. Therefore, the minimum sequence on Aβ that is required for its binding to heparin tetrasaccharide should be V12HHQKL17.

Biomolecule-based drugs are becoming a major force in the pharmaceutical industry. However, unlike the boost in protein-based drugs, the development of carbohydrate-based drugs is somewhat frustrated, mainly because of their heterogeneous structures and unspecific or unclear functions. Heparin oligosaccharides exhibited efficient anti-Aβ aggregation activity. However, these negatively charged and heterogonous carbohydrates are known to bind hundreds of endogenous proteins, which increases the risk of many side effects such as bleeding. GAGs are proven to have defined sequences. (Ly et al., 2011). Much as the discovery of a specific anti-thrombin pentasaccharide sequence in heparin has led to discovery of Arixtra, a successful synthetic anticoagulant drug. The Aβ binding sequence of heparin oligosaccharides elucidated herein provides a promising template for chemical or bio-synthesis of therapeutic agents for anti-Aβ aggregation. It could minimize undesired side effects while retaining Aβ binding activity and cross the blood-brain barrier. In addition, combining with efficient biosynthesis of GAGs (Xu et al., 2011) will accelerate the breakthrough of carbohydrate drugs in the new era of health care.

## Data Availability

The raw data have been deposited on Mendeley Data, and publicly available at https://data.mendeley.com/datasets/zmrvtt2296/1.
