# Supplementary material for: Characterizing Heparin Tetrasaccharides Binding to Amyloid-Beta Peptide
Source: Front Mol Biosci. 2022 Feb 23;9:824146. doi: 10.3389/fmolb.2022.824146 (PMC8906399; doi:10.3389/fmolb.2022.824146)
Supplement: Supplementary file 1 [file DataSheet1.docx]

***Supplementary Material***

**Preparation of heparin tetrasaccharides**

Heparin was partially digested with heparinase I. The oligosaccharides were fractionated using a GE AKTA purifier 100 system with a Superdex TM Peptide 10/300 GL column. The fractions were eluted with 0.2 M NH_4_HCO_3_ at a flow rate of 0.4 mL/min. The tetrasaccharide fraction was then separated using a Spherisorb S5 SAX 5 μm column. A linear gradient of 0-2.0 M NaCl (pH 3.5) over 2 h was used. The flow rate was 4 mL/min. UV absorption was monitored at 232 nm (Figure S1).

**
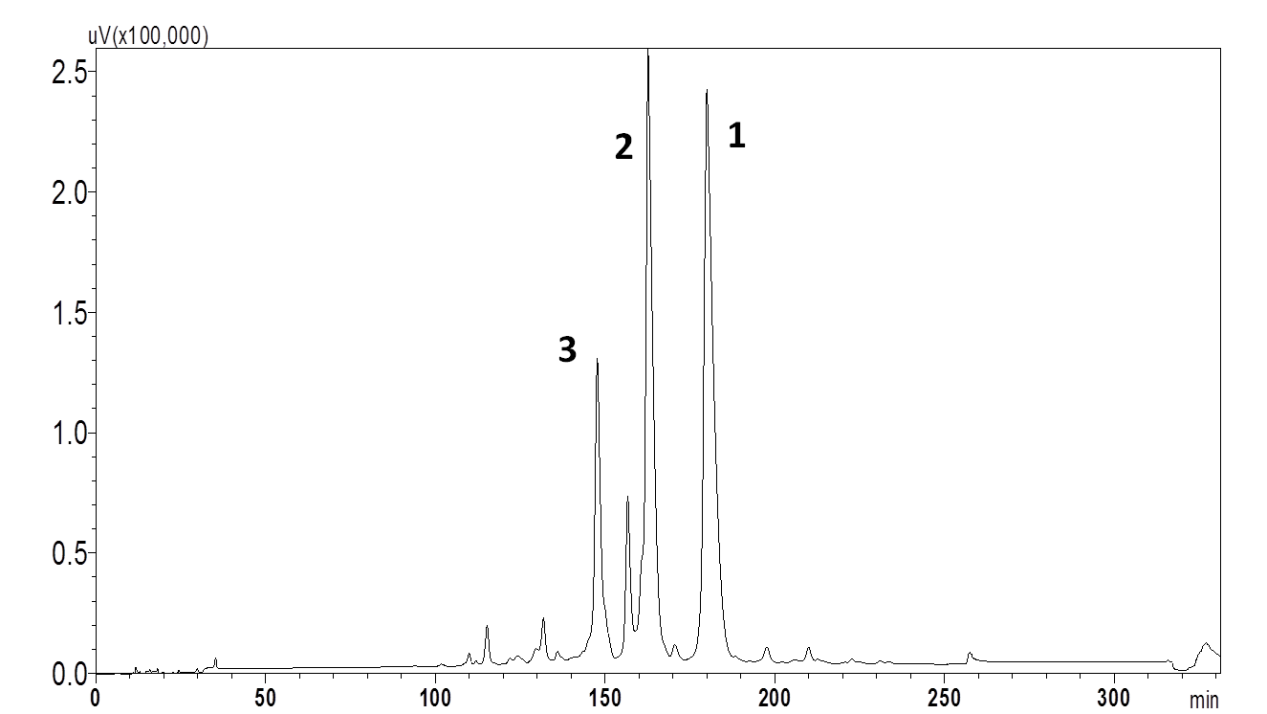
Figure S1.** Separation of tetrasaccharides **1-3** using strong anion-exchange chromatography.

**Structure characterization of oligosaccharides by MS/MS**

MS/MS studies were performed on an ion-trap-time-of-flight (IT-TOF) hybrid mass spectrometer (Shimadzu) in the negative mode. The samples were injected using a syringe in 50:50 (v:v) methanol/water containing 1 mM NaOH. The instrument parameters were set as follows: interface voltage, -3.5 kV; nebulizing gas flow rate, 1.5 L/min and collision-induced dissociation energy: 50%. The sequence was elucidated by glycosidic fragmentations and cross-ring fragmentations (Figure S2).

**
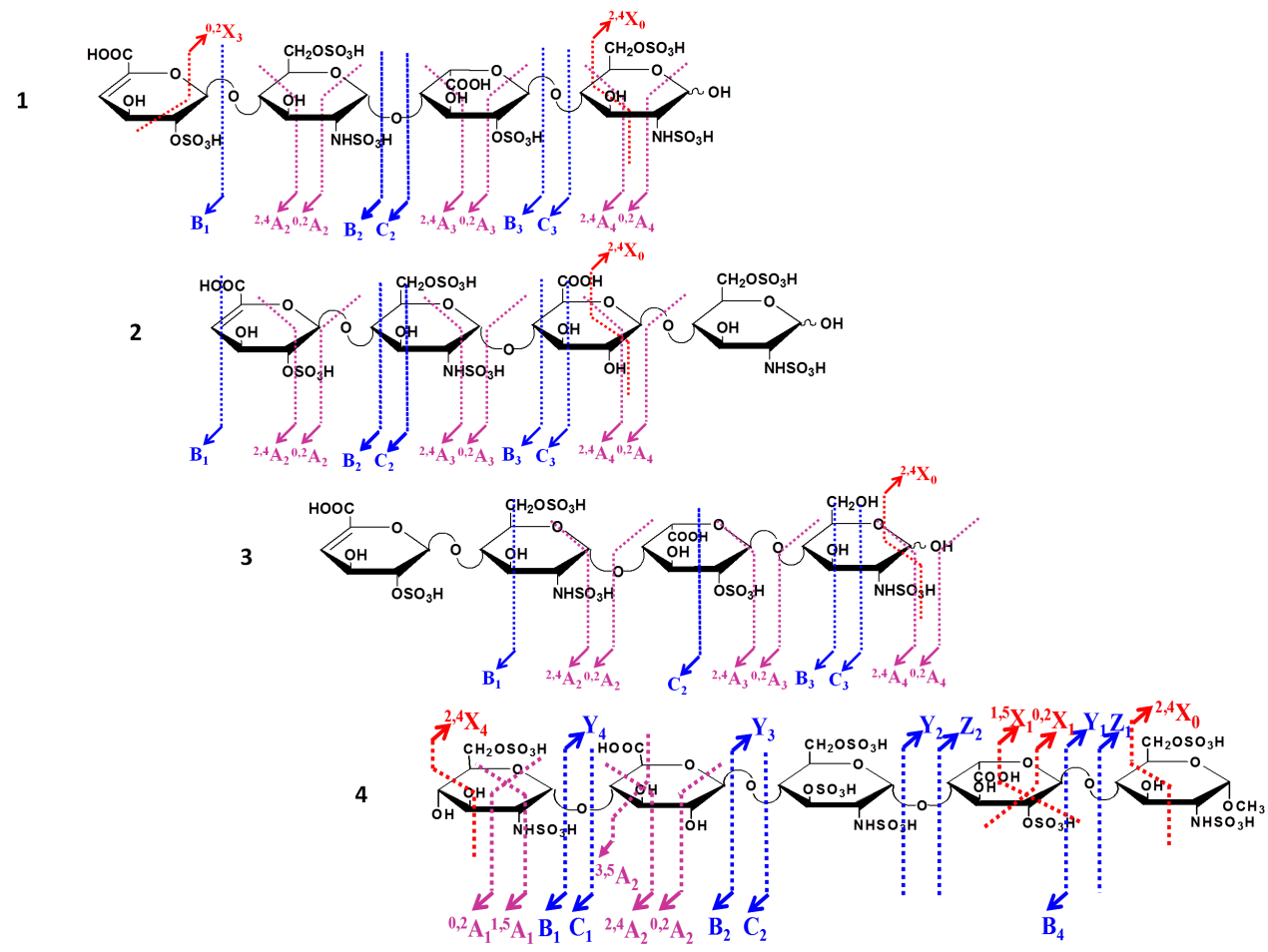
Figure S2.** MS/MS fragmentation of heparin oligosaccharides.

**Structure characterization of oligosaccharides by 2D-NMR**

3 mg of each oligosaccharide was dissolved in 500 μL of deuterium oxide (D_2_O) and lyophilized to remove exchangeable protons. The powder was then re-dissolved in 550 μL of D_2_O and transferred into 5 mm NMR tubes. NMR spectra were acquired at 600 MHz (^1^H) or 150 MHz (^13^C) with a Bruker AVANCE III 600 MHz spectrometer equipped with a 5 mm cryoprobe. Chemical shifts were recorded with reference to the HDO solvent signal at 298 K.

^1^H spectra were used to characterize the purity based on the signal integration (Figure S3).


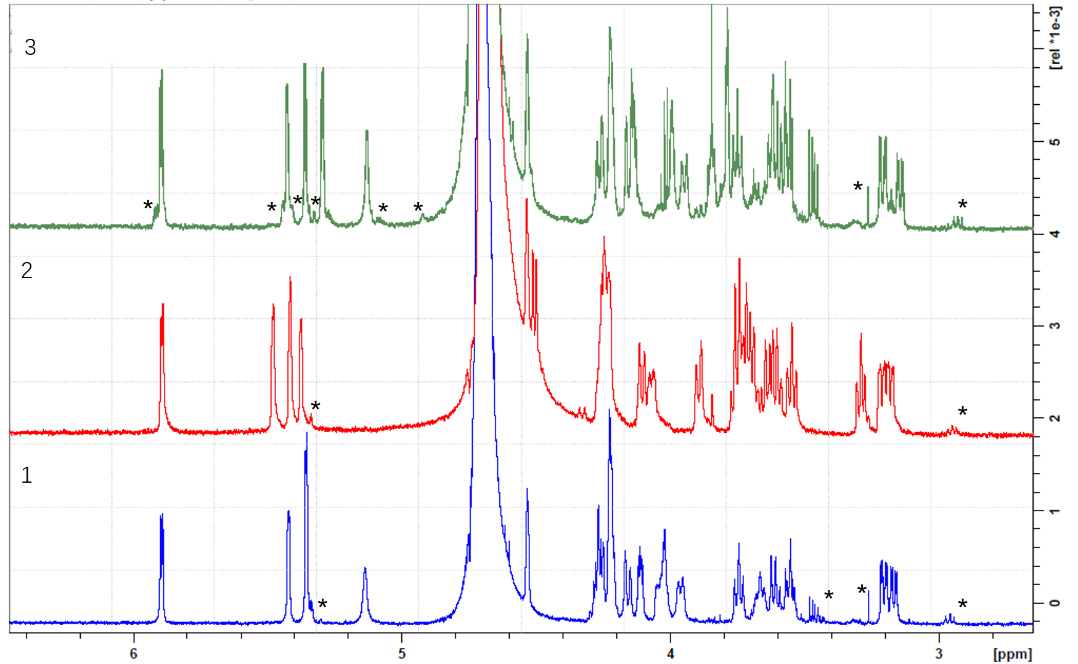


**Figure S3.** ^1^H spectra of heparin tetrasaccharides **1-3**. Impurity signals are labelled as asterisk.

2D ^1^H-^1^H COSY, ^1^H-^13^C HSQC and ^1^H-^13^C HMBC were acquired with standard pulse sequences. Cross peaks in the HSQC spectra of each oligosaccharide were assigned and labeled (Figure S4).

**
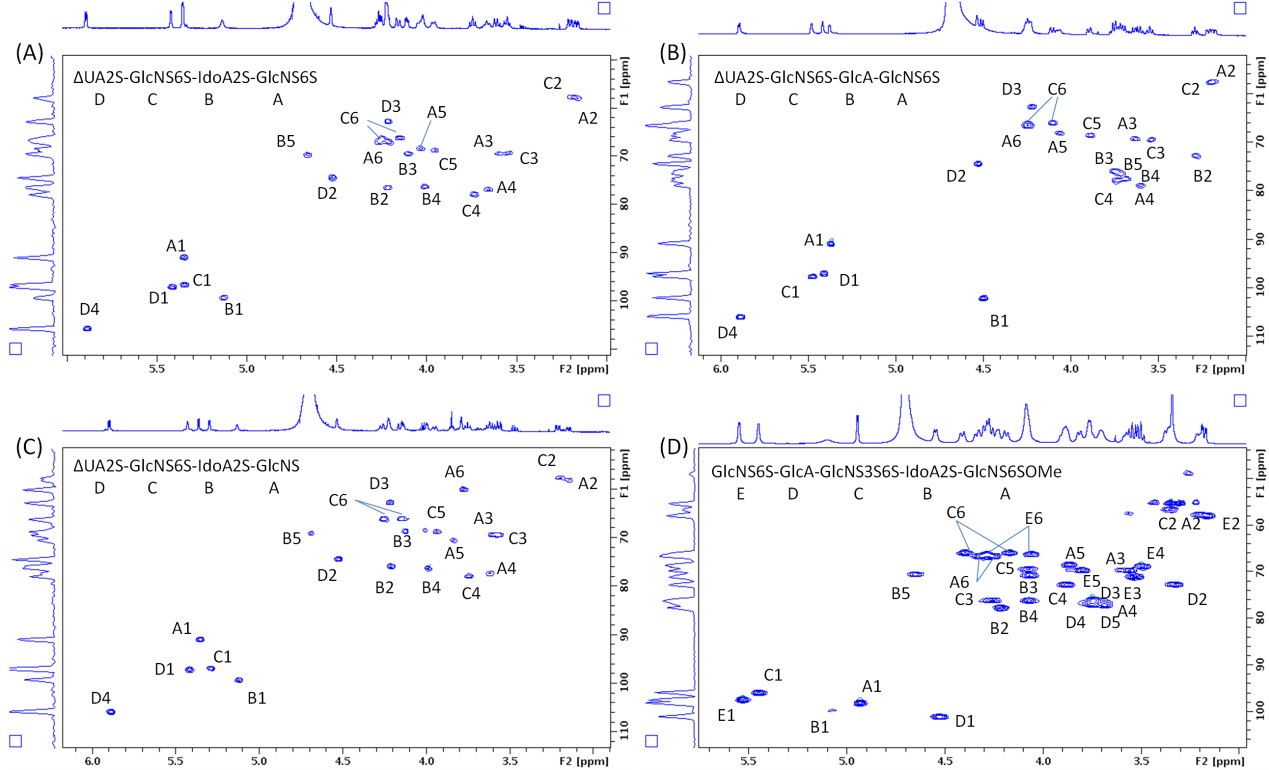
Figure S4.** ^1^H-^13^C HSQC spectra of heparin oligosaccharides. (A) **1**; (B) **2**; (C) **3** and (D) **4**.

**Molecular docking and modeling simulations**

Molecular docking and modeling were performed using AutoDock 4.2.6, a fully automatic docking program available open source. The solid-state NMR structure of Aβ was retrieved from the Protein Data Bank (PDB) under code 6TI5, which is a fibril of hexadecylmer. All of the hydrogen atoms were added to Aβ and Gasteiger charge was used. A volume of (120, 120, 120) grid points with 0.514 Å spacing was used. Aβ was put in the center of the cube larger enough to cover the whole surface of Aβ. The tetrasaccharide **1** was placed randomly into the box. During the whole docking process, all monosaccharide rings were fixed at their starting conformations, while the ring substituents were defined as flexible and could be rotated freely. Genetic Algorithm was used for docking.

**
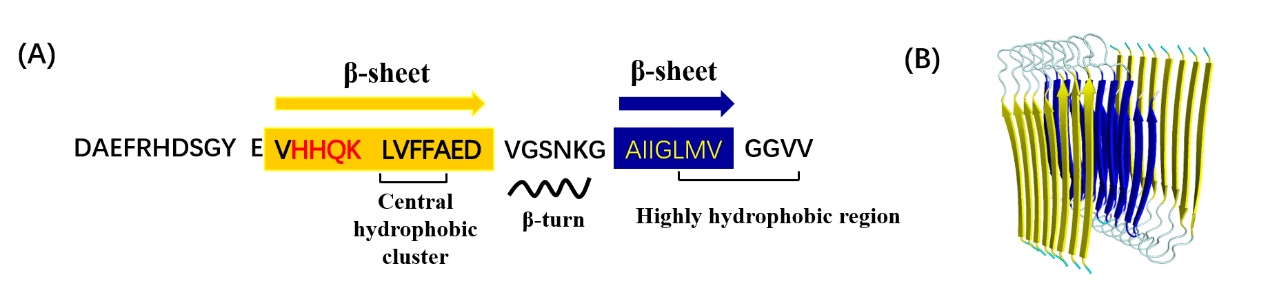
**

**Figure S5.** Structure of Aβ. (A) Amino acid sequence; and (B) 3D conformation of 6TI5.

**GMSA assay**

The GMSA assay was performed according to procedures previously described. Briefly, 1 mg Aβ was dissolved in 500 μL NaAc (20 mM, pH 3.5, precooled in ice) without any disaggregation process and the final concentration of 2 μg/μL Aβ was used thereafter. 2-aminoacridone-labeled tetrasaccharides were combined with Aβ in a certain molar ratio for 15 min at room temperature in a total volume of 20 μL. They were then loaded in wells of 1% agarose gel in 10 mM Tris-HCl (pH 7.4) and 1 mM EDTA. Electrophoresis was performed at 100 V for 20 min in a horizontal agarose electrophoresis system using an electrophoresis buffer comprising 40 mM Tris/acetic acid (pH 8.0) and 1 mM EDTA. The fluorescent oligosaccharides were visualized on a FluorChem Q gel analysis system.

**HDX-MS analysis**

An in-house system was set up using a Waters Acquity-H-CLASS UPLC and Xevo G2 Q-TOF mass spectrometer. A refrigerator was used to cool the injector, loop, columns, 6-way valve and switch valve. Aβ was treated in TFA and HFIP to break up any possible preexisting aggregates. The complex of Aβ and tetrasaccharide **1** was incubated in H_2_O (pH 3.5) at 4 °C for 120 min. Nine volumes of D_2_O (pH 3.5) were added to initiate the HDX. After 10 min, 1 μL of 10% ice-cold formic acid (FA) solution (pH 2.5) was added to quench the HDX. The deuterated Aβ was loaded onto an on-line pepsin proteolytic column. After digestion, the valve was switched, and the peptides flowed into a C18 column (5 μm, 2.1 × 50 mm). The mobile phase A was 0.1% FA, and mobile B was 0.1% FA in acetonitrile. The elution gradient was 20-60% mobile phase B in 6 min. The flow rate was 200 μL/min. The mass spectrometer parameters were set as follows: capillary voltage, 3 kV; source temperature, 100 °C; desolvation temperature, 350 °C. The spectra were acquired in MS^e^ mode with low collision energy of 6 V and high collision energy of 25-50 V. The data were analyzed by HX-Express software based on the centroid of the molecular ion isotope peaks. The deuterium percentage D was defined as:

where m_HDX_ is the molecular mass of the deuterated peptides, m_control_ is the molecular mass of non-deuterated peptides, N is the number of amino acid residues, and n_Pro_ is the number of proline residues.

**MTT assay**

The SH-SY5Y and PC 12 cell lines were cultured separately in Dulbecco's modified Eagle's medium supplemented with 10% fetal bovine serum at 37 °C, 5% CO2 in an incubator. The cells (4000 or 6000 cells/well) were seeded in 96-well plates and cultured in DMEM with 10% FBS. The culture medium was changed to fresh medium supplemented with 2 μM Aβ peptide alone or with **1** at concentrations of 10, 50 or 100 μg/mL. After 24 or 36 h incubation, 20 μl of 5 mg/mL MTT was added. After 4 h incubation at 37 °C, the supernatant was discarded, and 150 μL of DMSO was added. Enzyme-linked immunoassay was used to measure the absorbance value at 570 nm for the determination wavelength and 630 nm for the reference wavelength. Statistical significance was established by SPSS 17.0 statistical software, and single factor analysis of variance and P-values were represented as follows: *P < 0.05 and **P < 0.01. The experimental results were in the form of the mean and standard deviation.

**Interaction of Aβ with heparin oligosaccharides**

Heparin oligosaccharide was dissolved in D2O, into which Aβ was added, and the supernatant was transferred into the NMR tube. Complexes of heparin oligosaccharides and Aβ were prepared with a molar ratio of 1:1 in 120 μL of D_2_O. ^1^H-^13^C HSQC spectra were acquired. The overlaid HSQC spectra of **1-4** only and with Aβ are shown in Figure S6.


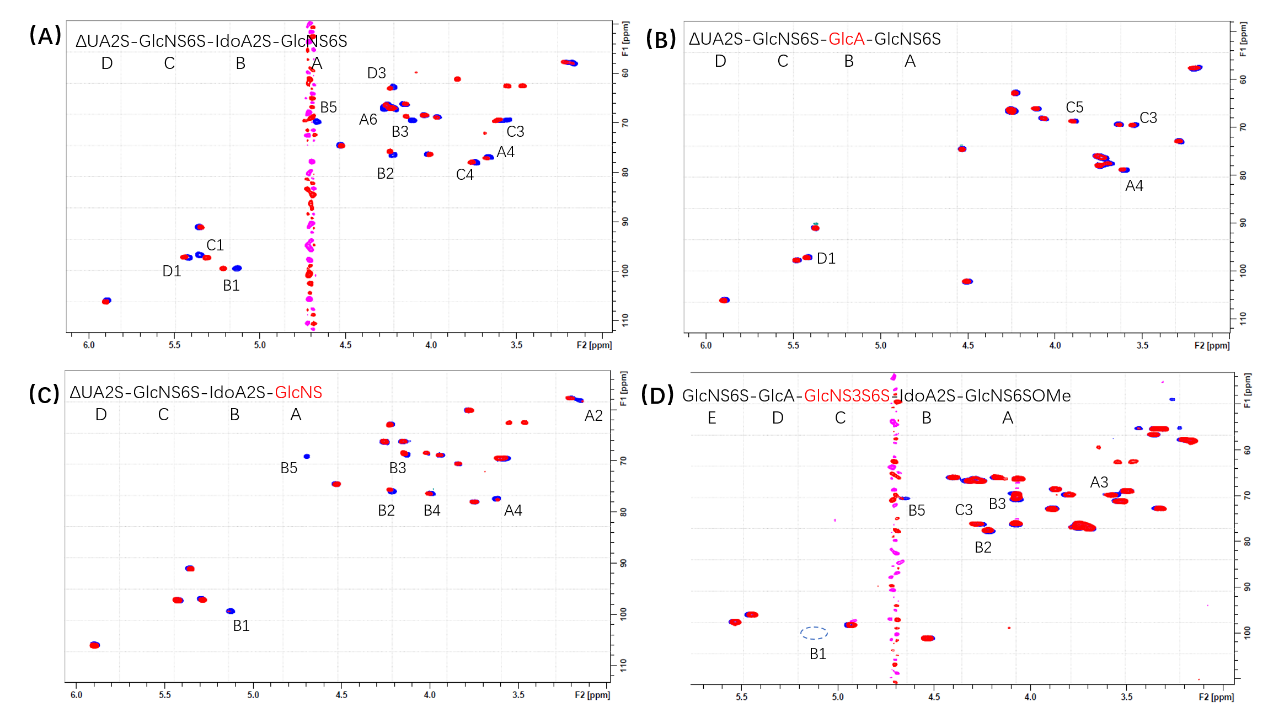


**Figure S6.** The overlaid ^1^H-^13^C HSQC spectra of **1-4** only (blue) and with Aβ (pink).
